# Near-infrared fluorescent imaging for parathyroid identification and/or preservation in surgery for primary hyperparathyroidism

**DOI:** 10.3389/fendo.2023.1240024

**Published:** 2023-12-04

**Authors:** Arslan Y. Pannu, Magdalena R. O’Connor-Manson, Lynda Wyld, Saba P. Balasubramanian

**Affiliations:** ^1^ University of Sheffield, Sheffield, United Kingdom; ^2^ Chesterfield Royal Hospital NHS Foundation Trust, Sheffield, United Kingdom; ^3^ Sheffield Teaching Hospitals NHS Trust, Sheffield, United Kingdom

**Keywords:** primary hyperparathyroidism, hyperparathyroidism, autofluorescence, near-infrared fluorescence, parathyroid glands, endocrine, surgery

## Abstract

**Introduction:**

Near infrared autofluorescence (NIRAF) is a novel intraoperative technology that has shown promising results in the localisation of parathyroid glands (PGs) over the last decade. This study aimed to assess the potential utility of NIRAF in first time surgery for primary hyperparathyroidism (PHPT).

**Methods:**

An observational study over a period of 3 years in patients who underwent surgery for PHPT was designed. Data on the use of NIRAF and fluorescent patterns in different organs (thyroid and parathyroid) and parathyroid pathology (single versus multi-gland disease) were explored. In addition, cure rates and operating times were compared between the NIRAF and no-NIRAF groups to determine the potential value of NIRAF in this cohort.

**Results:**

In 230 patients undergoing first time surgery for PHPT, NIRAF was used in 50 patients. Of these 50 patients, NIRAF was considered to aid parathyroid identification in 9 patients (18%). The overall cure rate at 6 months of follow-up was 96.5% (98% in NIRAF and 96.1% without NIRAF; p=1.0). The median (interquartile range) operating time was longer in the NIRAF arm at 102 minutes (74-120 minutes) compared to the no-NIRAF arm at 75 minutes (75-109 minutes); however, this difference was not statistically significant (p=0.542). Although the median parathyroid to thyroid (P/T) auto-fluorescence (AF) ratio was similar between single gland and multi gland disease (2.5 vs to 2.76; p=1.0), the P/T AF ratio correlated negatively with increasing gland weight (p=0.038).

**Conclusion:**

The use of NIRAF resulted in some potential “surgeon-perceived” benefit but did not lead to improvements in cure rates. The negative correlation between fluorescent intensity and gland weight suggests loss of fluorescence with pathology, which needs further investigation. Further studies on larger cohorts of patients, in depth analysis of fluorescence patterns between normal, adenomatous, and hyperplastic glands and evaluation of user experience are needed. Primary hyperparathyroidism, hyperparathyroidism, autofluorescence, near-infrared fluorescence, parathyroid glands, endocrine, surgery.

## Background

Primary hyperparathyroidism (PHPT) is characterised by elevated serum calcium, elevated or inappropriately high PTH levels due to autonomous parathyroid overactivity (1) and normal or high urinary calcium excretion (2, 3). The estimated incidence of primary hyperparathyroidism (PHPT) ranges between 0.4 and 21.6 cases per 100,000 individuals per year (4–6) with prevalence of 3 per 1000 in the general population (7). It is more common in women compared to men (3:1) and in the elderly (8). Currently, in the western world, most patients diagnosed with PHPT are asymptomatic and detected on routine testing of serum calcium (9–11); in contrast with the predominantly symptomatic presentation in underdeveloped countries. Surgery is the mainstay of treatment for most patients with PHPT; although conservative management is considered appropriate for some patients with mild, asymptomatic disease in the absence of end organ damage (12, 13).

The need for and extent of localisation of abnormal parathyroid glands prior to first time surgery for PHPT has been extensively debated. The traditional view of imaging is summarised in this quote: ‘the best tool for localisation of the PG is an experienced endocrine surgeon’ (14, 15). A UK survey demonstrated that 90% of surgeons rely on ultrasound (USS) and Sestamibi (MIBI) scans for preoperative localisation of PGs (16). The combination of USS and MIBI scans improves localisation of PGs compared to either technology used individually (17–19).

Despite the availability of numerous preoperative localisation techniques, appropriate identification of abnormal PGs is still challenging, particularly in patients with multi-gland disease and small adenomas. Intraoperative techniques like frozen section (FS) may help distinguish PGs from other structures such as lymph nodes, thyroid nodules, and brown adipose tissue with high accuracy (99.2% in a study of 1579 patients) but it cannot differentiate between normal and abnormal parathyroid tissue (20). Intraoperative parathyroid hormone (IOPTH) assays have also been used by surgeons to assess the adequacy of resection and as an alternative to FS. As with FS, this technique does not enable identification of parathyroid glands. A survey conducted in 2007 of the members of BAETS (British Association of Endocrine & Thyroid Surgeons) highlighted FS and IOPTH as the most widely used intraoperative tools (16).

Parathyroid tissue emits autofluorescence (AF) in the near-infrared spectral range of 700–900 nm when excited by a 785 nm diode laser. The fluorophore responsible for this AF has not been identified yet (21, 22). The diagnostic ability of AF in the detection of normal and pathological PGs ranges from 76 to 100% (21–28). A range of optical devices evaluate fluorescence intensity in the target tissue and are categorised as either image or probe based. PTeye™ (Medtronic®) is the only available probe-based device approved in the US and can identify tissues as either PG or not (29). Image-based systems on the other hand offer the advantage of providing a wide surgical view and the ability to visualise PGs in context to the surrounding anatomical structures, which is not possible with probe-based devices. However, image-based devices may not provide real time quantification of AF intensity.

Although early phase, single cohort studies have shown that early parathyroid identification during a neck exploration is possible and that normal and disease glands could be differentiated using AF, this has not yet been shown to improve clinical outcomes such as cure rates, operating times, and hypoparathyroidism in patients undergoing parathyroid surgery. In parathyroid surgery, early identification of abnormal glands may reduce the duration of surgery and early identification of normal glands may reduce need for unnecessary dissection in their search. This may in turn reduce operating times and risk of hypoparathyroidism.

The study aimed to assess the potential utility of NIRAF in surgery for PHPT by performing a detailed evaluation of a cohort of patients undergoing NIRAF imaging and comparing some clinical outcomes with a cohort of patients where NIRAF has not been used.

## Methodology

A retrospective, observational cohort study to evaluate the feasibility and clinical utility NIRAF in patients undergoing surgery for primary hyperparathyroidism. Comparison was made between two temporal cohorts: one from 2019 - 2022 (before the introduction of NIRAF) and the second between 2021 - 2022 (after introduction of NIRAF).

The project was registered as an NHS service evaluation by the host trust (No 11314) and local university ethics approval was obtained (No 051421). All adult patients undergoing surgery for biochemically confirmed PHPT over 3 years (July 2019 to June 2022) at a single UK teaching hospital were considered for inclusion. All patients had preoperative localisation studies to enable a decision on unilateral or bilateral neck exploration. All procedures were performed by, or under the direct supervision of, consultant endocrine surgeons. Patients undergoing reoperative neck surgery and those with renal HPT were excluded. Outcomes in this study included cure rates (i.e., normalisation of serum calcium and PTH levels after surgery), operating times, compared to a historical cohort of patients where NIRAF was not used and fluorescence patterns of normal and abnormal glands. Fluorescent patterns were correlated with pathology (single and multigland disease) and preoperative imaging. Patient demographics, preoperative biochemistry, radiology, relevant surgical, histology and follow up information were collected.

Surgery was performed as an open neck exploration in the standard manner.

All patients had a preoperative consultation with surgeons in an outpatient clinic. The preparation for surgery included thyroid function and voice assessment, as per routine practice in the unit. All neck explorations were performed via a transverse cervical incision made 2-3 cm above the sternal notch. The platysma muscle was divided, and strap muscles were separated in the midline. Following mobilisation of the thyroid gland and separation from the overlying strap muscles and the carotid sheath laterally, landmarks such as the inferior thyroid artery (ITA) and the recurrent laryngeal nerve (RLN) were identified. The surgeon then attempted to localise both parathyroid glands with a naked eye examination.

The EleVision™ IR Platform (Medtronic®) was introduced in March 2021 for use in parathyroid surgery to aid the surgeon in identification of parathyroid glands. After initial assessment of the operating field with naked eye examination, the Elevision™ camera was used at approximately 20 cm above the surgical field (after the operating room lights were turned off). In a small proportion of patients (n=5), a different fluorescent imaging device (Fluobeam LX™) was used. If NIRAF was used at surgery, the observations made by the surgeon, with regards to whether AF aided parathyroid identification, were recorded from the operative notes. The number of glands identified in each central compartment by naked eye (and by AF) were recorded for each patient. Excision of enlarged glands was carried out as per standard practice. Postoperatively, patients were observed for wound issues, voice change or swallowing problems. Patients had calcium (Ca^+2^), PTH, urea and electrolytes (U&E) and magnesium (Mg^+2^) levels checked after surgery. A cure was defined as the normalisation of adjusted calcium after surgery without hypercalcaemia (adjusted calcium >2.2 mmol/L) occurring in the first six months after surgery. The gland was considered abnormal if it weighed 100 mg or more or if patient was cured after excision of a single gland.

Recorded videos and images with EleVision™ were analysed with VisionSense™ (Medtronic®) software to assess AF intensity. The recorded videos can be reviewed in four different formats: visible (white) light, fluorescence mode and two other modes of fused images. Each video was examined to select clear images of PG that could be used for measurement of AF intensity. Six readings were recorded, each from the background thyroid and parathyroid glands. The highest value of the six parathyroid readings was recorded as peak fluorescent intensity.

A second observer then reviewed the images independently to identify the glands and record a second set of data on fluorescence intensity of the thyroid and parathyroid glands. If there was a discrepancy in identification of the organs (either thyroid or parathyroid) between the researcher and observer, it was settled by discussion.

Anonymised data was transferred to IBM SPSS® for statistical analysis (version 28.0). Descriptive data were reported using frequencies or percentages for categorical data, mean and standard deviation for parametric data and median and interquartile range (IQR) for non-parametric quantitative data. Inferential methods were based on the data type and distribution. A comparison of different outcomes between the different surgical approaches was performed using Chi-Square and Mann-Whitney U tests. A two-sided p-value of less than 0.05 was considered statistically significant. To perform reliability analysis, the mean AF readings for the thyroid and parathyroid glands were used to compute parathyroid to thyroid fluorescent intensity ratios (P/T AF ratio) and the agreement between observers was assessed using intraclass correlation coefficient (ICC).

## Results

Of the 250 patients who had surgery for parathyroid disease over a 3-year period, 20 were excluded (6 patients with renal HPT and 14 who underwent reoperative surgery). Of the remaining 230 patients who underwent first time surgery for PHPT, 56 (24.3%) were males and the median (inter-quartile range) age in years was 60 (52-70). The median (inter-quartile range) preoperative adjusted calcium and PTH levels were 2.71 (2.63-2.86) mmol/L and 13.2 (9.3-20.0) pmol/L respectively. The median first postoperative adjusted calcium and PTH levels were 2.40 (2.30-2.54) mmol/L and 1.5 (1.0-2.2) pmol/L respectively. Of these patients, 111 (48.3%) had a targeted or unilateral neck exploration (UNE) and 119 (51.7%) had a bilateral neck exploration (BNE). Intraoperative PTH assay (IOPTH) was used in 72 (31.3%). Preoperative imaging included ultrasound in 225 (97.8%) patients (of whom 69.7% had positive localisation) and Sestamibi in 215 (93.4%) patients (of whom 60.9% had positive localisation). The overall cure rate at 6 months of follow-up was 96.5% (98% in NIRAF and 96.1% without NIRAF; p=1.0). The median (inter-quartile range) length of stay was 1 (1-1) day.

NIRAF was used in 50 patients. Of these, the operating surgeon considered NIRAF to be beneficial in identifying PGs in 9 patients (18%). Among these 9 patients (of whom seven underwent unilateral neck exploration and two underwent bilateral exploration), AF helped in identification of PGs that were not initially seen with the naked eye in 7 cases and while in 2 cases, AF enhanced confidence in confirming the presence of PGs suspected on initial naked eye examination. In 7 of these 9 patients, the parathyroid glands identified with AF were not excised; underscoring how the technology assists in identifying and preserving normal glands. A comparison of cure rates, operating times, number of PGs identified intraoperatively and the discrepancy rate between intraoperative PG identification and histology in the NIRAF and no-NIRAF groups are shown in **Table 1**.

**Table 1:** Comparison of cure rates, operating times, PG identification and correlation with histology in patients undergoing surgery for primary hyperparathyroidism with NIRAF and without NIRAF.

	NIRAF (n=50)	No-NIRAF (n=180)	p-value
Cure rate (%)	49 (98.0%)	173 (96.1%)	1.0*
Median (IQR) operating times in minutes	102 (74 - 120)	92 (75 - 109)	0.542*
Median (IQR) glands identified (n)	2 (2 – 4)	2 (2 – 4)	0.097*
Discrepancy rate between surgical findings and histology	3 (6%)	13 (7.2%)	1.0*

*Fisher’s exact test.

IQR-inter-quartile range; NIRAF-Near infrared autofluorescence.

Out of a total of 16 discrepancies noted between intraoperative PG identification and histology, 13 were in the No-NIRAF group. The remaining 3 were in the NIRAF group. Due to small numbers, this difference was not statistically significant. However, in the BNE cohort, it was 4% in the NIRAF group and 10.7% in the no-NIRAF group. The operating times were longer when AF was used during surgery, although not statistically significant and without any impact on the number of glands identified or cure rates.

**Figure 1:**
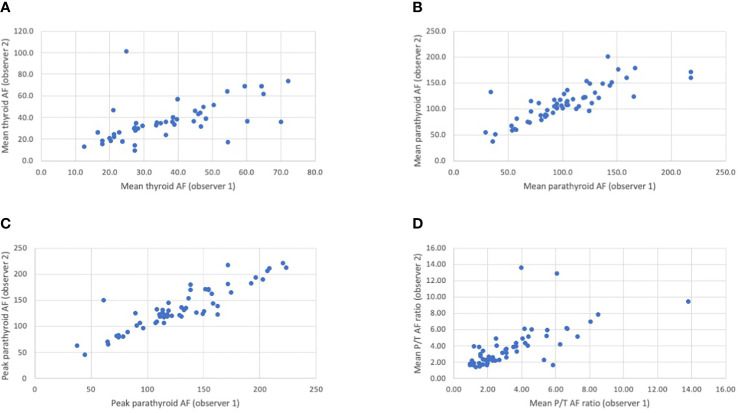
**(A–D)** Scatter plots showing the correlation between the two observers in the assessment of mean thyroid autofluorescence **(A)**, mean parathyroid autofluorescence **(B)**, peak parathyroid autofluorescence **(C)** and the mean P/T autofluorescence ratio **(D)**. The Intraclass Correlation (ICC) value for the mean thyroid, mean parathyroid and peak parathyroid autofluorescence (AF) intensities between observer 1 and observer 2 was 0.755, 0.899, and 0.896, respectively, indicating good agreement. The ICC value for the mean parathyroid/thyroid (P/T) autofluorescence (AF) readings between observer 1 and observer 2 was 0.667 indicating moderate agreement. *The ICC is a value between 0 and 1. <0.50: poor agreement; 0.50-0.75: fair agreement; 0.75 -0.90: good agreement; >0.90: excellent agreement.*.

Of 45 patients where Elevision™ was used, 133 glands were identified by the surgeon. Of these, video recordings of the fluorescence from only 58 glands (26 patients) were of adequate quality for further analyses. There was good agreement in the assessment of the mean background thyroid fluorescence, mean parathyroid fluorescence, and peak parathyroid AF intensities between two observers (intraclass correlation coefficient of 0.755, 0.899, and 0.896, respectively). The agreement on the ratio of mean parathyroid to mean thyroid (P/T) AF was moderate (0.667). These are illustrated in the **Figures 1A–D**. The average P/T AF ratio was then compared between normal and abnormal glands; single and multi-gland disease; glands positive and negative for MIBI uptake; and correlated with gland weight.

**Figure 2:**
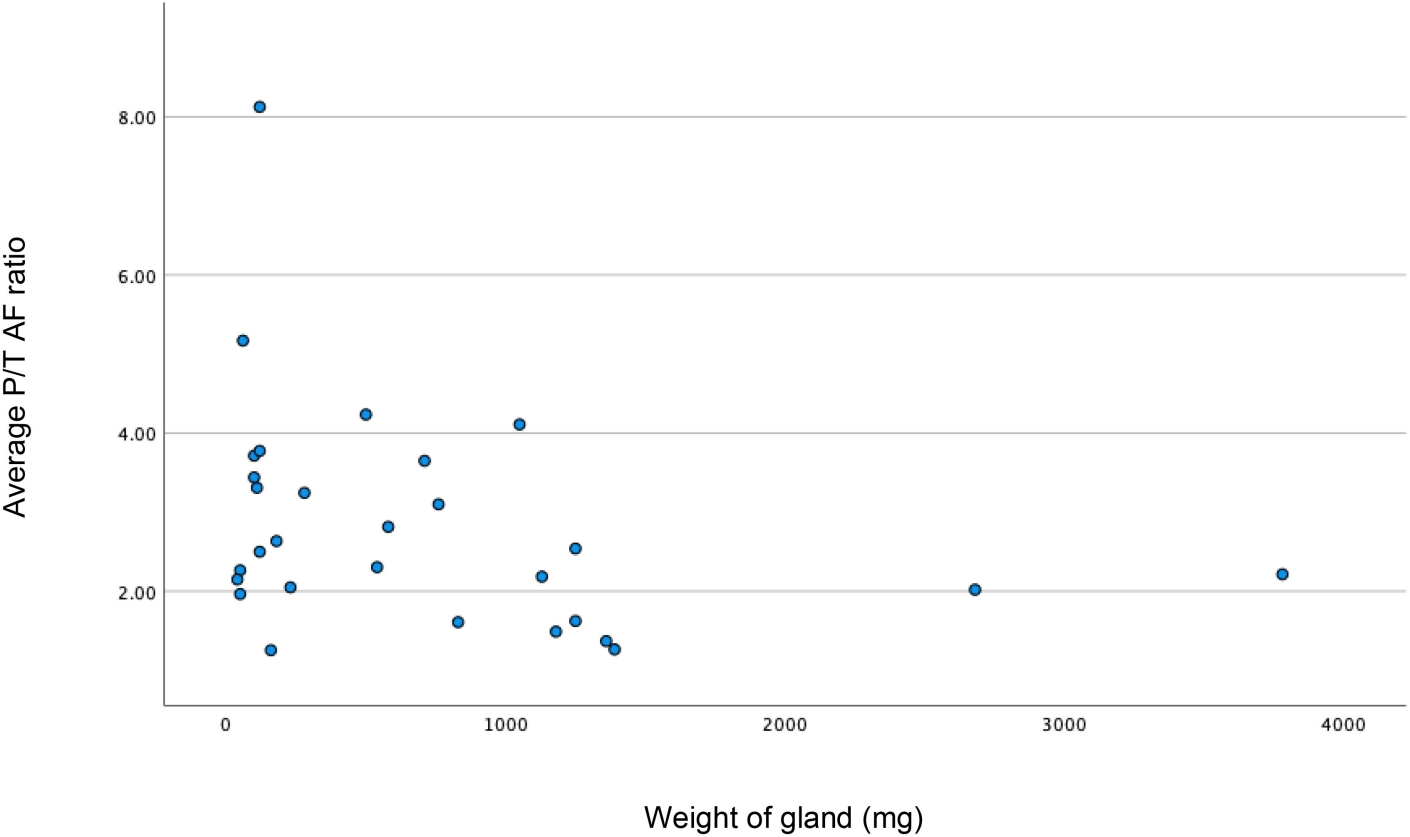
Scatter plot showing weak correlation between increase in weight (milligrams) of the gland resulting decrease in P/T (parathyroid/thyroid) autofluorescence (AF) ratio. *Spearman’s rho = -0.338; p<0.038; n=29)*.

The median (IQR) parathyroid to thyroid AF ratio of 33 normal glands was 3.63 (2.03-5.47); compared to 2.5 (1.82-3.54) in 25 abnormal glands (p=0.88; Mann-Whitney test). The median (IQR) fluorescence for 10 glands in the multi gland disease (MGD) group was 2.76 (1.62-3.66), compared to 2.5 (2.05-3.24) for the 19 glands in the single gland disease (SGD) group (p=1.0; Mann Whitney U test). The median (IQR) P/T AF ratio was 3.21 (1.92-5.20) in MIBI negative glands (n=45) and 2.30 (2.02-3.10) in MIBI positive glands (n=11); again, this was not statistically significant (p=0.197). **Figure 2** shows the correlation between gland weight and P/T AF ratio; showing that the intensity of fluorescence seems to decrease with increasing gland weight (p=0.038).

**Figure 3:**
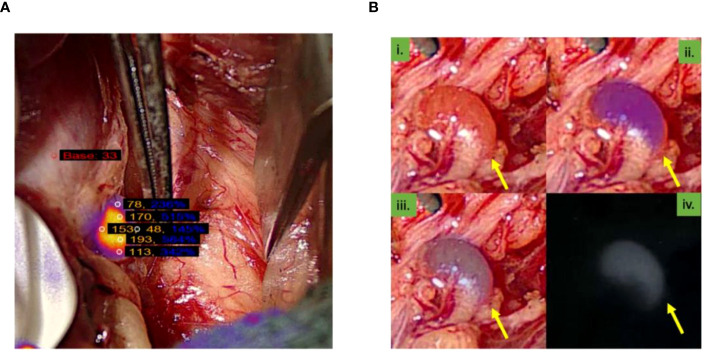
**(A)** Image from the VisionSense^TM^ software showing six autofluorescence readings obtained from the parathyroid gland; **(B)** Four images of different modes of normal parathyroid gland obtained with the use of EleVision^TM^ IR platform (Medtronic®). Yellow arrow point towards parathyroid gland.

**Figure 4:**
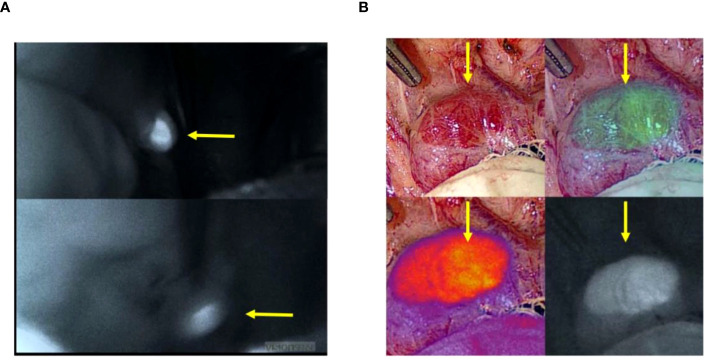
**(A)**. Higher autofluorescence intensity seen in the normal (upper left picture) than abnormal (lower left picture) parathyroid glands; **(B)**. ‘Fluorescence cap’ seen in an abnormal parathyroid gland. Yellow arrow point towards parathyroid gland.

Six autofluorescence readings obtained from the parathyroid gland with the help of the VisionSense™ software are shown in **Figure 3A** while, four different modes of a normal parathyroid gland obtained with the use of EleVision™ IR platform (Medtronic®) are shown in **Figure 3B**. Intense autofluorescence was seen in the normal PG than the abnormal (**Figure 4A**) and ‘fluorescence cap’ as described in the literature is demonstrated for one or the abnormal PG (**Figure 4B**).

## Discussion

This is the first UK study to investigate the role of parathyroid AF by EleVision™ in patients undergoing surgery for PHPT. The device provides both a qualitative signal and allows for fluorescence intensity measurements. Like other image-based devices, it uses a diode laser light with an excitation wavelength of 785 nm, and emitted fluorescence between 800 – 850 nm is captured using a specialised camera system and presented on a monitor in the context of surrounding tissues (30).

The cure rate in this study was over 96%, which is consistent with the typical cure rate described in the literature (31). In this study, normal glands exhibited brighter and more intensive fluorescence signals compared to abnormal (enlarged) glands; consistent with previous studies (23, 32). In one study, the lower intensity AF in abnormal glands was associated with increased cellularity, clusters of Oxyphil cells, patchy fibrosis, and haematoma in the excised gland(s) (32).

In this cohort, in patients undergoing bilateral neck exploration (BNE), the discrepancy rate between intraoperative findings and histology in the NIRAF and no-NIRAF patients were 4.0% and 10.7%, respectively. Bilateral neck exploration is typically used when preoperative imaging is inconclusive or in patients suspected to have MGD. On average, an equal number of glands were found with and without the use of AF in this study. However, overall, autofluorescence helped in the identification of PGs that were not initially visible to the naked eye in 7 patients, while in 2 cases, AF confirmed the presence of PGs suspected on initial naked eye examination. This amounts to 18% (95% CI of 9.5% and 31%) of patients where NIRAF was used. It remains to be seen if this number is similar in other studies and whether this ‘surgeon perceived’ benefit does translate to improvement in clinical outcomes such as cure rates, hypoparathyroidism and operating times. Other studies have shown that use of NIRAF intraoperatively can increase the number of identified PGs (25, 33), help identify PGs early (34). Increasing the number of PGs identified seem to be associated with reduced rates of parathyroid auto-transplantation and lower rates of postoperative hypocalcaemia (35).

The data also demonstrated that as the weight of the gland increased, intensity ratio of the parathyroid and thyroid gland decreased (p=0.038). This confirms findings of other studies, which have explained that larger glands produce less intense fluorescence (32, 36). As larger glands are easier to identify, AF may not be necessary to identify these glands, but relevant to identification and preservation of normal glands.

A higher average P/T AF ratio was seen in MGD patients, but this was not statistically significant. This could be explained by the presence or excision of normal glands in this group. Squires and colleagues observed low intensity of AF in patients with MEN1 and PHPT as compared non-MEN1 cohort (37). Another study by same authors also reported no significant difference in the intrinsic AF between hypercellular adenomas and normocellular PGs (38). AF intensity may well vary in different disease processes causing HPT and this needs further exploration.

In this study, MIBI negative patients had higher fluorescence intensity; but this was not statistically significant, probably due to small numbers. It was previously believed that mitochondria, which are rich in NADPH and more abundant in oxyphil cells, might be responsible for the fluorophore associated with AF. DiMarco and colleagues however found no correlation between MIBI positive glands and AF intensity (39). It is possible that the fluorophore responsible for AF in PGs may express itself in different ways, resulting in specific fluorescence patterns for normal, adenomatous, and hyperplastic glands, but this theory has not been proven yet (24, 32).

Assessment of fluorescent intensities may be subjective and influenced by variation in fluorescence in different parts of the same gland and the background. The thyroid was therefore used as the denominator to calculate parathyroid to thyroid fluorescent ratios. In addition, two observers recorded fluorescent intensities to assess agreement between observers and increase the reliability of this assessment. These analyses showed that the agreement (ICC) for mean background thyroid, mean parathyroid and peak parathyroid AF intensities between observer 1 and observer 2 were 0.755, 0.899, and 0.896, respectively. The lower agreement in AF intensity in the thyroid can be attributed to the inherent heterogeneity of thyroid fluorescence and differences in site of recording of fluorescence. The excised glands were not assessed with AF in this study, as the data obtained was inconsistent and of poor quality, and therefore not suitable for AF assessment.

The use of AF during surgery was found to result in increased operating time without affecting cure rates, which is a common drawback when introducing new technology in clinical settings. The increased operating time could be attributed to time in setting up the AF device in the theatre and the interruption caused by switching the room lights off and on (21, 23, 24). However, with increased use of fluorescence imaging, the use of AF devices is expected to become more streamlined, resulting in reduced operating and theatre times.

The identification of PGs with preoperative imaging relies on glands being enlarged or abnormal. Large glands are easy to visualise, and AF may not be necessary for identification of these glands. AF is more appropriate in these settings to identify normal glands which need to be preserved and to differentiate them from a lymph node or abnormal thyroid nodule.

The study was a retrospective, single-centre study with limitations inherent to this study design including small sample size and lack of long term follow up. Several interesting associations between fluorescent patterns and imaging and pathology could not be shown to be statistically significant, due to small sample sizes. While the EleVision™ device was considered easy to interpret, it required the surgeon to hold the device steady at a constant distance from the operating field, which could result in operator fatigue and shaky images; resulting in the unavailability of good quality images for many patients. This cohort included patients in the learning curve for this technology and the perception of its usefulness evolved over the course of the study. The technology was not used in consecutive patients and was based on availability and surgeon preference. The lack of standardisation in the use of this technology also limits the internal validity of the results. However, the assessment has been done in a pragmatic fashion as is often the case with early evaluation of technology in ‘real life’. This study also made the assumption that all glands left *in situ* were normal.

Despite these limitations, this study has demonstrated the potential value of an image based fluorescent detection device (Elevision™) in surgery for primary hyperparathyroidism. The significant negative correlation between gland weight and fluorescent intensity is an important clinical observation, that reinforces the findings of other recent studies. In addition, despite the small cohort, the technology was considered beneficial during surgery in a significant proportion of patients (18%); suggesting its value in difficult cases.

Further research in larger cohorts of patients and randomized controlled trials involving multiple centres with long-term follow-up are necessary to establish the effectiveness and safety of AF in various clinical scenarios, such as identifying PGs, differentiating between normal and abnormal glands, reducing postoperative complications like hypocalcaemia and hypoparathyroidism, and evaluating the impact of surgeon experience on AF use.

## Data availability statement

The raw data supporting the conclusions of this article will be made available by the authors, without undue reservation.

## Author contributions

All authors listed have made a substantial, direct, and intellectual contribution to the work and approved it for publication.
